# Potential Anti-Infectious Activity of Essential Oil Chemotypes of *Lippia origanoides* Kunth on Antibiotic-Resistant *Staphylococcus aureus* Strains

**DOI:** 10.3390/plants13091172

**Published:** 2024-04-23

**Authors:** Andrés Humberto Uc-Cachón, Luz María Calvo-Irabien, Angel de Jesús Dzul-Beh, Haziel Eleazar Dzib-Baak, Rosa Grijalva-Arango, Gloria María Molina-Salinas

**Affiliations:** 1 Unidad de Investigación Médica Yucatán, Instituto Mexicano del Seguro Social, Mérida 97150, Yucatán, Mexico; andresuccachon@gmail.com (A.H.U.-C.); angeldzulbeh1992@gmail.com (A.d.J.D.-B.); hazieldzibbaak@gmail.com (H.E.D.-B.); 2 Unidad de Recursos Naturales, Centro de Investigación Científica de Yucatán, Mérida 97204, Yucatán, Mexico; rgarango@cicy.mx

**Keywords:** *Lippia origanoides*, essential oil, chemotypes, *Staphylococcus aureus*, antimicrobial resistance, antibiofilm, antihemolysis

## Abstract

*Staphylococcus aureus* infections are prevalent in healthcare and community environments. Methicillin-resistant *S. aureus* is catalogued as a superbug of high priority among the pathogens. This Gram-positive coccus can form biofilms and produce toxins, leading to persistent infection and antibiotic resistance. Limited effective antibiotics have encouraged the development of innovative strategies, with a particular emphasis on resistance mechanisms and/or virulence factors. Medicinal aromatic plants have emerged as promising alternative sources. This study investigated the antimicrobial, antibiofilm, and antihemolysis properties of three different chemotypes of *Lippia origanoides* essential oil (EO) against susceptible and drug-resistant *S. aureus* strains. The chemical composition of the EO was analyzed using GC-MS, revealing high monoterpene concentrations, with carvacrol and thymol as the major components in two of the chemotypes. The third chemotype consisted mainly of the sesquiterpene β-caryophyllene. The MIC values for the two monoterpene chemotypes ranged from 62.5 to 500 µg/mL for all strains, whereas the sesquiterpene chemotype showed activity against seven strains at concentrations of 125–500 µg/mL, which is the first report of its anti-*S. aureus* activity. The phenolic chemotypes inhibited biofilm formation in seven *S. aureus* strains, whereas the sesquiterpene chemotype only inhibited biofilm formation in four strains. In addition, phenolic chemotypes displayed antihemolysis activity, with IC_50_ values ranging from 58.9 ± 3.8 to 128.3 ± 9.2 µg/mL. Our study highlights the importance of *L. origanoides* EO from the Yucatan Peninsula, which has the potential for the development of anti-*S. aureus* agents.

## 1. Introduction

The discovery of antibiotics is one of the most significant advances in medicine [1]. However, the overuse and misuse of antibiotics have become prevalent among outpatients, resulting in drug resistance and ending the gold era of antibiotics [2]. Antimicrobial resistance (AMR) is one of the principal public health problems of the 21 century and threatens the effective prevention and treatment of diverse infections caused by parasites, fungi, bacteria, and viruses that are not susceptible to the common drugs used to treat them [3]. AMR in bacteria is particularly urgent because, in recent decades, bacterial resistance to new antibiotics has developed in both community and healthcare-associated infections [4]. In 2017, the World Health Organization published a list of global priority microbes, including 12 species of bacteria with critical, high, and medium antibiotic resistance, and called upon academic and pharmaceutical scientific communities to conduct investigations to fight AMR [5]. One of these species is methicillin-resistant *Staphylococcus aureus* (MRSA), a Gram-positive coccus that spreads in the healthcare environment and community and causes a variety of infections such as suppurative or abscessed lesions, surgical wound infections, bacteremia, pneumonia, osteomyelitis, arthritis, and intravascular catheter-associated infection or urinary catheterization. The main serious complications of staphylococcal bacteremia are septic shock, endocarditis, myocarditis, meningitis, and pneumonia [6].

Numerous in vitro biological studies have been performed using commercially available reference strains, and only a few have included clinical isolates. It is important to perform a bioactivity assay using priority drug-resistant clinical isolates that involve mechanisms such as biofilm-formation and toxin production, which are associated with recalcitrant infections, to test a possible new antibiotic against wild/circulating bacteria [7,8]. Currently, the search for novel therapies for treating *S. aureus* infections is a prevalent area of research, with a particular emphasis on resistance mechanisms and/or virulence factors. Essential oils (EOs) obtained from aromatic plant species have emerged as a promising alternative [9,10,11,12].

Oregano is a spice commonly used in various culinary traditions and has been found to have medicinal properties. There are approximately 60 plant species in the oregano ethnobotanical complex, primarily from the genera *Origanum* and *Lippia*, which are characterized by the presence of carvacrol and thymol. *Lippia origanoides* (botanical synonyms *L. graveolens* and *L. sidoides*) is considered an American oregano species [13,14]. In traditional medicine, dried or fresh *L. origanoides* leaves have been used to treat respiratory and gastrointestinal diseases, such as colds, bronchitis, and diarrhea. It is also used to treat uterine inflammation, menstrual cramps, postpartum cramps, vaginal infections, headaches, wounds, mycoses, and pain [15,16,17,18]. Recently, EOs from *L. origanoides* have shown antimicrobial activity, including a reduction in biofilm-formation and antiquorum sensing (QS) activity [12,19,20].

EOs are obtained from aromatic plants via steam distillation and consist of lipophilic, low-molecular-weight volatile compounds, including monoterpenes, sesquiterpenes, phenylpropanes, and oxygenated derivatives [21]. The effectiveness of these compounds against pathogens is influenced by their EO composition and is primarily attributed to the most abundant metabolites present at high concentrations, which can exhibit simple, additive, or synergistic effects. The chemical complexity of EOs, which target multiple bacterial cell types simultaneously, reduces the likelihood of resistance in microorganisms [22,23,24,25,26].

The chemical composition of EOs depends on various factors, including the internal and external plants factors. Chemotypes, which are individuals from a plant species with an identical morphology that present EOs with different chemical compositions, are often found in aromatic species [27]. The *Lippia origanoides* EO has significant variability in its chemical composition, with two main phenolic chemotypes characterized by the oxygenated monoterpenes, carvacrol and thymol, and a non-phenolic chemotype with a higher diversity of metabolites [28,29,30].

To gain a more comprehensive understanding of the antimicrobial activity of the native *L. origanoides* Kunth EO from the Yucatan Peninsula, which is traditionally used to treat fever, asthma, cough, diarrhea, stomach cramps, and pain [31], and explore its potential against resistant strains of *S. aureus,* we evaluated three EO chemotypes against reference strains and clinical isolates of *S. aureus* including methicillin-resistant *S. aureus* (MRSA) and vancomycin-resistant *S. aureus* (VRSA). In this study, we analyzed the effects of these chemotypes on bacterial growth, drug resistance mechanisms, and virulence factors.

## 2. Results

### 2.1. Chemical Composition of the Three Chemotypes of the L. origanoides EO

The chemical composition of the *L. origanoides* EO varied considerably among the three chemotypes. The two phenolic chemotypes were dominated by monoterpenes and oxygenated monoterpenes (<80% chromatogram area), with carvacrol and thymol, respectively, as the major components, representing >55% of the total chromatogram area. In contrast, for the sesquiterpene chemotype, in which sesquiterpenes represented 80% of the total chromatogram area, β-caryophyllene, α-humulene, and α-selinene were the most abundant sesquiterpenes (Table 1).

### 2.2. Activity of the L. origanoides EO on Staphylococcal Growth

The antibacterial activity of the *L. origanoides* EO depends on the interaction between the bacterial strain and the chemotype. The antibacterial activities of the three chemotypes are summarized in Table 2. Clinical isolates (except SAU-UIMY-44) were found to be more susceptible to the three chemotype EOs than all the ATCC reference strains.

In total, 41.6% of the *S. aureus* strains (clinical isolates: SAU-UIMY-1, SAU-UIMY-10, SAU-UIMY-24, SAU-UIMY-26, and SAU-UIMY-39) were the most susceptible to the EO of the phenolic chemotypes (MIC = 62.5–125 µg/mL), while only in 16.6% (ATCC 25923 [500 µg/mL] and SAU-UIMY-5 [250 µg/mL]) of *S. aureus* strains was the activity of the three chemotypes equal. The SAU-UIMY-16 clinical isolate was the most susceptible to the three EO chemotypes (62.5–125 µg/mL). The carvacrol and thymol chemotypes showed MIC values between 62.5 and 500 µg/mL for all strains. In the case of the sesquiterpene chemotype, this EO only exhibited activity (125–500 µg/mL) on seven strains: SAU-UIMY-16, SAU-UIMY-31, SAU-UIMY-5, SAU-UIMY-10, SAU-UIMY-39, ATCC 25923, and SAU-UIMY-24 (Table 2). The EO of the three chemotypes showed bactericidal effects on various *S. aureus* strains, except for SAU-UIMY-44 (Table 2).

### 2.3. Activity of the L. origanoides EO on the Formation of the Biofilm of S. aureus

The effect of *L. origanoides* EO chemotypes on biofilm formation inhibition coincided with the results of the antibacterial assays. This response depends on the interaction between the *S. aureus* bacterial strain and EO chemotype. A two-way ANOVA showed a statistically significant interaction between the bacterial strain and EO chemotype (F_(16,54)_ = 20.73, *p* = 0.001) on the inhibitory effect on biofilm formation. Phenolic chemotypes inhibited biofilm formation with IC_50_ values of less than 250 µg/mL in all *S. aureus* strains (except for SAU-UIMY-26 and SAU-UIMY-44). In contrast, the sesquiterpene chemotype presented an IC_50_ biofilm-inhibition value lower than 250 µg/mL for the strains SAU-UIMY-1, SAU-UIMY-31, SAU-UIMY-39, and *S. aureus* MRSA ATCC 4330. In these four strains, the effect of the sesquiterpene chemotype EO was statistically equal to that of phenolic chemotypes (Table 3). For the EO of the three chemotypes, a negative correlation was observed between the IC_50_ values of the EO on biofilm formation and the biofilm biomass (OD values) of the various *S. aureus* strains. The Spearman rank correlation coefficient (R) values were −0.73 *p* = 0.025; −0.85 *p* = 0.004; and −0.93 *p* = 0.0003 for the carvacrol, thymol, and sesquiterpene chemotypes, respectively. The clinical isolate SAU-UIMY-1 presented the highest biofilm biomass and the lowest IC_50_ values of the sesquiterpene (IC_50_ = 30.8 ± 1.5), thymol (IC_50_ = 60.6 ± 1.7), and carvacrol (IC_50_ = 77.3 ± 1.7) EOs. A similar trend was observed for the SAU-UIMY-31 and MRSA ATCC 43300 strains, with superior biofilm biomass values and generally lower IC_50_ values (Table 3).

### 2.4. Correlation of the Chemical Composition of the L. origanoides EO and Its Antibiofilm Activity

The nine *S. aureus* biofilm producing strains presented a significant association between the IC_50_ values for biofilm inhibition and the concentration (% peak area in chromatogram) of the major chemical components in the EO of *L. origanoides* chemotypes, as shown by the values of the Spearman rank correlation coefficient (Table 4). 

The inhibition of biofilm formation varied according to the *S. aureus* strain. In two clinical isolates, SAU-UIMY-1 and SAU-UIMY-31, the concentration of the sesquiterpenes β-caryophyllene, α-humulene, α and β selinene, and γ and δ cadinene were significantly correlated with the IC_50_ values. Higher concentrations of these metabolites in the EO resulted in lower IC_50_ values. In the remaining seven clinical isolates, the percentages of the monoterpenes, *p*-cymene, carvacrol, and thymol showed a significant association with biofilm inhibition (Table 4).

### 2.5. Antihemolysis Activity of the L. origanoides EO on S. aureus

**Figure 1:**
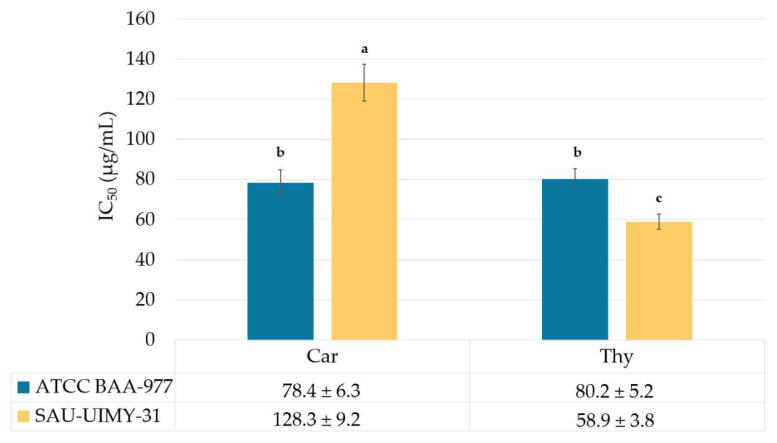
Antihemolysis activity of the *L. origanoides* EO on *S. aureus.* Car: carvacrol; Thy: thymol; average IC_50_ values (±SD) with the same letter showed non-significant differences in the post hoc Tukey test (*p* < 0.05). The sesquiterpene chemotype IC_50_ is >250 µg/mL for both strains.

In our study, the carvacrol and thymol chemotypes of the *L. origanoides* EO exhibited antihemolysis activity against *S. aureus* ATCC BAA-977 and SAU-UIMY-31. A two-way ANOVA showed a statistically significant interaction between the bacterial strain and the EO chemotype (F_[1,8]_ = 339.9, *p* = 0.0001) on the IC_50_ average value. Among them, the thymol chemotype in strain SAU-UIMY-31 displayed the best activity (IC_50_ = 58.9 ± 3.8 µg/mL), while in the carvacrol chemotype, the lowest IC_50_ value was observed in the reference strain ATCC BAA-977 (IC_50_ = 78.4 ± 6.3 µg/mL; Figure 1). On the other hand, the sesquiterpene EO chemotype did not exhibit 50% hemolysis inhibition at its highest concentration (250 µg/mL).

## 3. Discussion

### 3.1. Chemical Composition of the Three Chemotypes of the L. origanoides EO

The three chemotypes identified in this study for the *L. origanoides* EO have been previously reported for this species [20,28,29,32]. These chemotypes were also observed in *O. vulgare* cultivars [33].

### 3.2. Activity of the L. origanoides EO on Staphylococcal Growth

Previous studies have reported the antibacterial activity of the *L. origanoides* EO against *S. aureus* strains. Gómez-Sequeda et al. 2020 evaluated the antibacterial activity of the *L. origanoides* EO against *S. aureus* ATCC MRSA and reported MIC_50_ values of 600, 1600, and >3000 µg/mL for carvacrol, thymol, and non-phenolic chemotypes, respectively [20]. Likewise, Martinez et al. 2021 reported antibacterial activity against *S. aureus* ATCC 29213 (MSSA) of the *L origanoides* thymol–carvacrol chemotype (major components: thymol 32.7%, carvacrol 18.8%, β-caryophyllene 6.4%, γ-terpinene 5.2%, and *p*-cymene 1.1%). The results indicated that *L. origanoides* EO exhibited an MIC_50_ value of 450 µg/mL [34]. The higher MIC values obtained in this study could be attributed to higher carvacrol and thymol concentrations, as these authors reported concentrations lower than 35% for both phenols.

Our research found that the majority of *L. origanoides* EOs were bactericidal agents. This information provides valuable insights into the mechanisms of action of the antibacterial agents. However, no extrapolations have been made to clinical therapeutics. Previous studies have shown that carvacrol and thymol, as phytoconstituents of EO, as well as pure compounds, exhibit bactericidal activity against *S. aureus* [20,33,34,35]. To the best of our knowledge, this is the first report of the bactericidal activity of the sesquiterpene chemotype of *L. origanoides*.

### 3.3. Activity of the L. origanoides EO on the Formation of the Biofilm of S. aureus

Gómez-Sequeda et al. (2020) reported that the carvacrol and thymol chemotypes of *L. origanoides* exhibited values of IC_50_ = 70 µg/mL and 1200 µg/mL, respectively, against *S. aureus* ATCC MRSA biofilms, whereas the non-phenolic chemotype showed an IC_50_ > 3000 µg/mL against the same strain [20]. Martinez et al. 2023 reported that the *L. origanoides* EO (thymol 32.7% and carvacrol 18.8%) exhibited a biofilm inhibition of 72% at 400 µg/mL against *S. aureus* ATCC 29213 (MSSA). In addition, these authors reported that EO alters the expression of genes related to biofilm formation and virulence factors associated with the QS system [35]. Our study found similar results for the carvacrol and thymol chemotypes. It is important to highlight that the non-phenolic chemotype, with a predominance of sesquiterpenes (β-caryophyllene, α-humulene, and α-selinene), also showed significant biofilm inhibition activity, like that of the phenolic chemotype, particularly against the *S. aureus* strains SAU-UIMY-1, SAU-UIMY-31, SAU-UIMY-39, and MRSA ATCC 4330.

Our study demonstrated that higher biofilm biomass producers were inhibited by lower concentrations of EO, but the antibiofilm activity of EO against *S. aureus* strains may not be directly related to the type of biofilm biomass producers. Rather, it may depend on the unique genetic characteristics of each strain [36]. The accessory gene regulator (*agr*) QS system in *S. aureus* has been found to correlate with biofilm biomass. *Staphylococcus aureus* strains can be grouped into four categories based on their *agr* system (*agr*I–IV). These groups often exhibit different phenotypes, such as varying biofilm formation capacities [37,38]. Certain chemotypes of *L. origanoides* with thymol (32.7%) and carvacrol (18.8%) have been found to reduce the expression of *agr* in *S. aureus* [35], suggesting that differences in the type of *agr* among the strains could affect the biofilm biomass production and IC_50_ antibiofilm activity of EO.

The *Lippia origanoides* EO suppresses the *ica* operon (ADBC), which is a key regulator of polysaccharide intercellular adhesin (PIA) synthesis in *S. aureus* biofilms [39]; *icaA* and *icaD* are responsible for PIA synthesis [40]. Previous research has demonstrated that the co-expression of *icaA* and *icaD* in *S. aureus* is required for strong biofilm production; however, the absence of these genes does not completely inhibit biofilm formation, implying the existence of *ica*-independent mechanisms [39]. These differences may also contribute to discrepancies in antibiofilm activity among EO chemotypes.

### 3.4. Correlation of the Chemical Composition of the L. origanoides EO and Its Antibiofilm Activity

Our results found a relationship between the presence of β-caryophyllene, α-humulene, α and β selinene, γ and δ cadinene *p*-cymene and carvacrol and the prevention of biofilm formation. Some EO components have been reported to exhibit antibiofilm properties. Studies have frequently reported the antibiofilm effects of the monoterpenes carvacrol and thymol, particularly against *S. aureus* [9,41]. Reichling (2020) discovered that carvacrol and thymol significantly reduced biofilm mass and inhibited the movement of bacteria [9]. Similarly, Kim et al. (2022) found that applying carvacrol and thymol at sublethal concentrations can effectively combat *S. aureus* by impacting its ability to form biofilm [41]. Our findings align with those of previous studies, as the *L. origanoides* EO from the carvacrol and thymol chemotypes showed a significant inhibitory effect on biofilm formation. Carvacrol and thymol have been shown to impede biofilm development by impacting membrane lipids, preventing protein accumulation, and halting the microcolony stage [42,43,44]. It also affects cell viability and interacts with transcriptional regulators of QS communication, biofilm formation, and virulence genes [35]. The activity of EO components can be influenced by their minor components in complex interactions, which could explain the variations in the anti-*S. aureus* activity of the *L. origanoides* EO depending on the bacterial strain and EO composition. The monoterpene *p*-cymene may enhance the activity of other antimicrobial compounds such as carvacrol and/or thymol, which cause membrane instability [34,45,46]

The sesquiterpene β-caryophyllene exhibited anti-*S. aureus* activity on fusidic acid resistant to the strain [47], which induces apoptosis and the disruption of mitochondrial membrane potential [48] and, using molecular docking analyses, interacts with bacterial DNA gyrase B, suggesting that it acts as an inhibitor [49]. The biological activity of β-selinene against *S. aureus* ATCC 25923 has also been reported [50]. It has been shown that two or three major components, constituting up to 85% of the chromatogram area, drive biological activity [25,26]; however, often, the minor components also play a role [22,24]. Because of the diverse chemical nature of EOs, their components could exert additive, antagonistic, or synergistic effects on each other; therefore, the specific modes of action could be a result of these interactions. Detailed studies with specific experimental designs are needed to deepen our understanding of the interactions between the different metabolites present in the EO of *L origanoides* and their anti-*S aureus* effect. Our results provide a basis for designing and conducting such experiments in future research.

### 3.5. Antihemolysis Activity of the L. origanoides EO on S. aureus

Previously, the antihemolytic effects of the three chemotypes of the *L. origanoides* EO from Colombia have been reported: 1 (thymol 32.7% and carvacrol 18.8%), 2 (thymol 22.1% and 10.7% carvacrol), and 3 (without thymol or carvacrol) were 54%, 28%, and inactive, respectively. These results highlight the importance of thymol and carvacrol in antihemolytic activity [34]. An EO from *L. origanoides* (thymol 32.7%, carvacrol 18.8%) inhibits the expression of QS genes, including the synthesis of a transcriptional regulator RNA III, responsible for several virulence factors, such as α and δ hemolysins [35]. In addition, sublethal concentrations of carvacrol and thymol reduced hemolytic activity [41]. 

## 4. Materials and Methods

**Figure 2:**
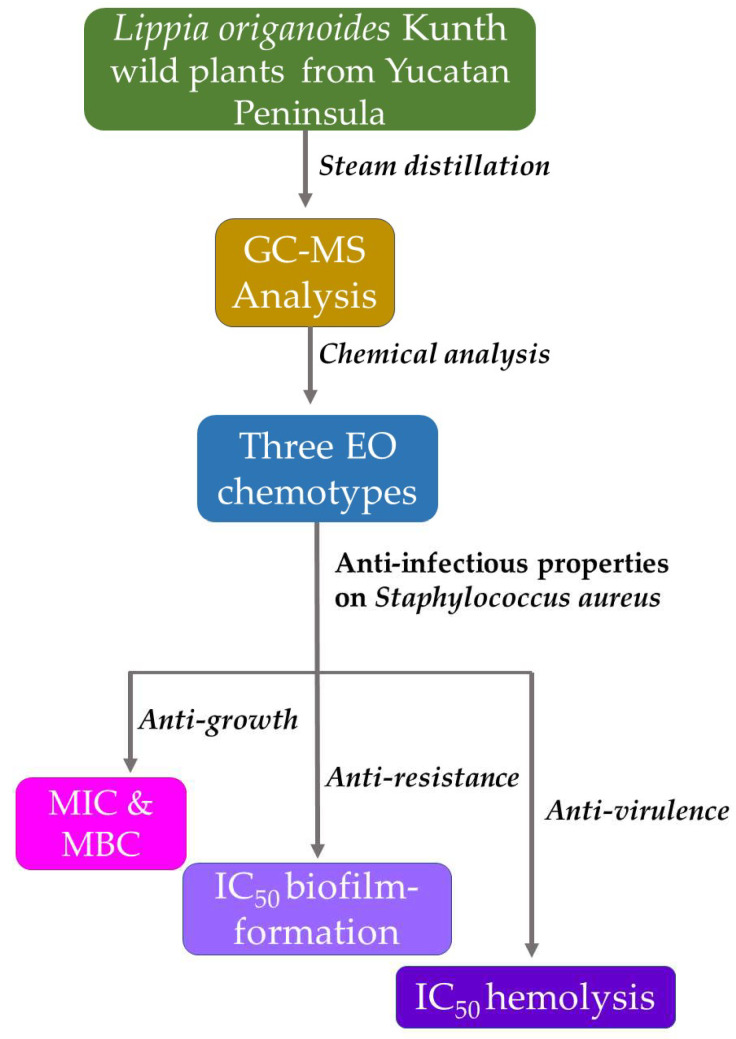
Flowchart of the methodology.

This project was approved by the Scientific and Ethics Committees National of the Instituto Mexicano del Seguro Social (IMSS), with approval number R-785-2022-009. Figure 2 shows the flowchart of the methodology.

### 4.1. EO Extraction and Chemical Characterization

*Lippia origanoides* leaves from adult plants were collected from wild populations located in the Yucatan Peninsula [51]. The chemotype carvacrol was collected in Chicxulub Puerto Yucatán (21.253° N 89.573° W); the thymol chemotype was collected in San Felipe Yucatán (21.561° N, 88.189° W); and the sesquiterpene chemotype was collected in Sotuta Yucatán (20.514° N 88.946° W). The leaves were dried at 35 °C for 36 h in an air-flux drying oven (NOVATECH HS60, Tlaquepaque, Mexico) and stored at 4 °C until distillation, following the method described in [51]. Herbaria voucher specimens were deposited at the Centro de Investigación Científica de Yucatán, Mérida, Mexico (LM Calvo 240, 248, and 258); species identification was performed by the herbaria staff. EO was extracted from 250 g of dried leaves using hydrodistillation in a Clevenger-type apparatus for 1.5 h, with 25:1 mL/g water: plant material and 44 drops per minute as the average distillation rate, with hexane as the collector solvent. The oil–hexane mixture was dried with sodium sulfate, and the solvent was evaporated under a flow of nitrogen. The samples were stored in sealed amber vials at 4 °C until biological assays and chromatographic analysis.

To analyze the chemical composition of the EO in the three *L. origanoides* chemotypes, GC-MS analyses were conducted using an Agilent 6890N gas chromatograph (Agilent Technologies, Santa Clara, CA, USA), which was connected to an Agilent 5975 mass-selective detector and equipped with G1701DA GC-MSD ChemStation software v.D.03.00.552. The non-polar DB5 5% phenyl-metilpolisiloxane column (60 m, 0.25 mm i.d., film thickness 0.25 μm) was used, with a temperature program of 45 °C for 5 min, 45 to 150 °C at 4 °C/min, 150 °C for 2 min, 150 to 250 °C at 5 °C/min, 250 °C for 5 min, 250 to 275 °C at 10 °C/min, and 275 °C for 15 min. The injector temperature was 280 °C and the detector temperature was 290 °C. The carrier gas was He (1.5 mL/min); the injection volume was 1 μL, and the split ratio was 1:40. Mass spectra (MS) were obtained by electron impact at an energy of 70 eV. The temperatures of the ionization chamber and the transfer line were maintained at 230 °C and 285 °C, respectively. MS, total ionic currents, and extracted ions were obtained with a quadrupole analyzer using automatic radiofrequency scanning (full scan) in the mass range of *m*/*z* 35–300.

The arithmetic linear retention index (AI) of each peak was determined [52], relative to that of a homologous series of n-alkanes (C8–C40), which was injected in the CG-MS under the same conditions previously described. For a metabolite to be considered tentatively identified, its mass spectrum (MS) would have to match the corresponding mass spectra contained in the library ADAMS 2007 [52] and in the library of the National Institute of Standards and Technology (NIST 11). In addition, the corresponding AI value must coincide with the AI values reported in the literature [52,53,54]. When available, the definitive identification of individual metabolites was carried out by comparing their MS with those of commercial standards such as carvacrol, thymol, β-caryophyllene, and eucalyptol (98–99% purity, Sigma-Aldrich, St. Louis, MO, USA) or authentic samples previously purified and identified in our laboratories. The quantification of metabolites was performed using a Varian 430 GC (Varian BV, Santa Clara, CA, USA) equipped with an FID detector and N_2_ as the carrier gas, using the same column, temperature program, and conditions described previously.

### 4.2. Anti-Infectious Assays

#### 4.2.1. *Staphylococcus aureus* Strains

Three reference strains and nine clinical isolates of susceptible and drug-resistant *S. aureus* were used in the bioassays (Table 5). All strains were maintained at −80 °C in tryptic soy broth (TSB; Becton Dickinson, Franklin Lakes, NJ, USA) supplemented with glycerol (J.T. Baker, Inc., Phillipsburg, NJ, USA).

#### 4.2.2. Activity of EO on Staphylococcal Growth

The Minimal Inhibitory Concentration (MIC) of EO against the *S. aureus* strains was determined using the resazurin microtiter assay (REMA) broth dilution method. Briefly, bacterial cultures were cultured on Mueller–Hinton agar (MHA; Becton Dickinson Co., Ltd., Franklin Lakes, NJ, USA). Then, two to three bacterial colonies were suspended in 3 mL of Muller–Hinton broth (MHB; Becton Dickinson Co., Ltd., Franklin Lakes, NJ, USA) and incubated at 37 °C for 2–4 h until reaching growth comparable to the turbidity of the 0.5 McFarland standard (DEN-1; Biosan, Riga, Latvia). This suspension was further diluted at 1: 50 to obtain the working inoculum, and 100 μL of this suspension was incubated and cultured with 100 μL of MHB containing the EO at serial dilutions ranging from 500 to 15.62 μg/mL. All assays included a positive control (cultures with antibiotic specific) and negative controls (culture-free wells of the EO or antibiotic (Sigma-Aldrich, St. Louis, MO, USA) and a sterility control (culture broth alone). After incubation at 37 °C for 16 h, 30 μL of resazurin (Sigma-Aldrich, St. Louis, MO, USA) was added, and the microplates were incubated again at 37 °C for 2 h. A well-defined pink color indicated positive bacterial growth, whereas a blue color indicated the absence of growth. The results were expressed as the MIC, which corresponded to the greatest dilution of the EO in which a color shift from blue to pink was not observed. Each assay was performed three times independently in duplicate [55].

The minimal bactericidal concentration (MBC) of the EO was determined by reseeding the bacterial culture according to the method described by de Jesús Dzul-Beh et al. 2023 [56]. Succinctly, 5 μL of the EO-treated bacterial suspensions corresponding to MIC, 2× and ½ MIC in MHB in 96-well plates, were transferred to a new microplate containing 195 μL of fresh culture medium per well. In addition, antibiotic-treated bacterial suspensions (positive control), untreated bacterial suspensions (negative control), and culture medium alone (sterility control) were transferred to fresh broth [57]. After incubation at 37 °C for 16 h, resazurin was added to the wells, as previously described. MBC corresponded to the minimal EO concentration that did not cause a color shift in cultures re-incubated in the fresh medium. Each assay was performed three times in duplicate, and the MBC/MIC index for the EO was calculated.

#### 4.2.3. Activity of the EO on the Formation of Biofilm

The inhibition of biofilm formation in all strains of *S. aureus* was evaluated using the crystal violet (CV) staining method in flat-bottom 96-well microplates, as previously described [55]. Briefly, the strains were activated and cultured on MHA, and two bacterial colonies were cultured in 3 mL of TSB (Beckton Dickinson Co., Ltd., Franklin Lakes, NJ, USA) at 37 °C for 24 h. Then, an aliquot was transferred into TBS supplemented with glucose 1% (*w*/*v*; TSB + G) to match the turbidity of 0.5 McFarland. This suspension was further diluted at 1:50, and 100 μL of this was incubated with 100 μL of TBS + G containing serial dilutions of 500 to 15.6 μg/mL concentrations of the EO. An ethylenediaminetetraacetic acid (Sigma-Aldrich, St. Louis, MO, USA) solution was used as the positive control, whereas the negative control was EO-free wells. The microplate was incubated for 24 h at 37 °C. After incubation, the culture broth was gently aspirated, and each well was washed thrice with sterile distilled water to remove non-adherent cells and dried at 60 °C for 45 min. The biofilm was stained by incubating for 30 min with 200 μL of 0.1% CV (Sigma-Aldrich, St. Louis, MO, USA) solution. Any excess CV was removed with sterile distilled water before adding 200 μL of 40% acetic acid (Fermont, Monterrey, Mexico), and the absorbance was measured at 490 nm using a microplate reader (IMark; Bio-Rad, Hercules, CA, USA), which was related to the amount of biofilm produced. Each assay was performed in triplicate, and the concentration of EO that inhibited 50% (IC_50_) biofilm formation was calculated using GraphPad Prism ver. 5 software (GraphPad Software Inc., La Jolla, CA, USA).

#### 4.2.4. Antihemolytic Activity of EO

The antihemolytic activity of the EO was assessed using *S. aureus* ATCC BAA-977 and the clinical isolate SAU-UIMY-31, according to Loges et al., 2020, with minor modifications [58]. Briefly, an aliquot on an overnight culture of *S. aureus* was transferred into fresh TSB to match the turbidity of 0.5 McFarland. A total of 30 μL of the suspension was incubated with 2970 μL of TBS containing serial dilutions of 200 to 25 μg/mL EO and incubated with shaking at 200 rpm and 37 °C for 24 h. After incubation, the bacterial culture supernatant was collected via centrifugation. In total, 100 μL of the bacterial supernatant was incubated with 300 μL of diluted human red blood cells in PBS buffer (Sigma-Aldrich; 330 μL red blood cells/10 mL PBS buffer), with shaking at 200 rpm and 37 °C for 1 h. The mixture was centrifuged at 4000 rpm for 4 min to remove intact red blood cells, and the absorbance of the supernatant was measured at 430 nm using a microplate reader (IMark, Bio-Rad, Hercules, CA, USA). The percentage of hemolysis inhibition for each concentration of EO was calculated. Each assay was performed in triplicate, and the IC_50_ hemolysis was calculated using GraphPad Prism ver. 5 software (GraphPad Software, Inc., La Jolla, CA, USA).

### 4.3. Statistical Analysis

The Spearman rank correlation between the IC_50_ for biofilm formation inhibition and the biofilm producer index was estimated for the nine biofilm producer strains. Similarly, for each bacterial strain, a Spearman rank correlation was estimated between the IC_50_ for biofilm formation inhibition and the concentration of the major components (% area in the chromatogram) in the three chemotypes of the *L. origanoides* EO (N = 9). Two-way ANOVA was used to analyze the interaction of the bacterial strain and EO chemotype composition on the IC_50_ values for biofilm formation inhibition and antihemolytic activity. In all statistical analyses, results with *p*-values > 0.05 were considered non-significant.

## 5. Conclusions

In the present study, the chemical composition of the three chemotypes of the *L. origanoides* EO varied considerably. The two phenolic chemotypes mainly contained carvacrol and thymol, whereas the third chemotype contained sesquiterpenes, with β-caryophyllene as the major component. All chemotypes exhibited anti-staphylococcal and antibiofilm activities against the reference and clinical isolates of *S. aureus* strains. The activity in both assays varied depending on the interaction between the bacterial strain and the EO chemotype. However, the carvacrol and thymol chemotypes also showed antihemolytic activity against reference and clinical isolates of *S. aureus* strains. Our study highlights the potential of chemotypes of the *L. origanoides* EO from the Yucatan Peninsula as candidates for future research in developing anti-*S. aureus* agents, and they are even active against the drug-resistant strains MRSA and VRSA.

## Figures and Tables

**Table 1:** Chemical composition of the EO chemotypes of *L. origanoides*.

Compound Name	Class	AI ^a^	AI ADAMS ^b^	Chemotype			Compound Identification ^d^
				Car	Thy	Ses	
				% area ^c^	% area ^c^	% area ^c^	
myrcene	M	992	988	1.15	2.15	0.34	RI, MS, Coi
p-cymene	M	1029	1020	**18.12**	**15.26**	2.85	RI, MS, Coi
limonene	M	1033	1024	0.25	0.41	1.06	RI, MS, Coi
eucalyptol	OM	1036	1026	0.74	2.63	2.77	RI, MS, Coi
γ-terpinene	M	1061	1054	1.30	0.24	0.39	RI, MS, Coi
thymol methyl ether	OM	1238	1232	1.31	3.33	0.07	RI, MS
thymol	OM	1295	1289	**2.74**	**57.30**	0.13	RI, MS, Coi
carvacrol	OM	1309	1298	**68.51**	0.20	0.19	RI, MS, Coi
α-copaene	S	1388	1374	nd	nd	3.29	RI, MS, Coi
β-caryophyllene	S	1438	1417	1.46	**7.75**	**33.00**	RI, MS, Coi
β-copaene	S	1445	1430	0.04	0.08	1.00	RI, MS
α-humulene	S	1471	1452	0.80	3.84	**13.42**	RI, MS, Coi
aromadendrene-allo	S	1479	1458	nd	nd	1.73	RI, MS
γ-muurolene	S	1491	1478	nd	nd	4.23	RI, MS
β-selinene	S	1502	1489	nd	0.13	5.05	RI, MS
α-selinene	S	1510	1498	nd	0.21	**7.91**	RI, MS
γ-cadinene	S	1529	1513	nd	nd	1.36	RI, MS
δ-cadinene	S	1539	1522	nd	0.08	7.21	RI, MS
caryophyllene oxide	OS	1600	1582	0.79	1.33	1.32	RI, MS, Coi
Total of compound class (%)							
M				20.82	18.06	4.64	
OM				73.3	63.46	3.16	
S				2.3	12.09	78.20	
OS				0.79	1.33	1.32	
Total identified (%)				97.21	94.94	87.32	

Only compounds with a >1% chromatogram area are shown. Car: carvacrol; Thy: thymol; Ses: sesquiterpene; M: monoterpenes; OM: oxygenated monoterpenes; S: sesquiterpenes; OS: oxygenated sesquiterpenes. ^a^ AI: Linear retention indices determined experimentally on the DB5 column relative to a series of n-alkanes (C8–C40). ^b^ Bibliographic linear retention indices. ^c^ Relative contents are given as GC-FID peak areas; for the three most abundant compounds of each chemotype, the contents (% area of chromatogram) are given in bold type; n.d. = not detected. ^d^ Identification method: RI = tentative identification based on AI; MS = tentative identification based on MS; Coi = co-injection with commercial standard GC-FID.

**Table 2:** Anti-*S. aureus* activity of the three chemotypes of the *L. origanoides* EO.

*S. aaureus* Strain	Drug-Resistant Profile	*L. origanoides* EO						Positive Control
		Car		Thy		Ses		
		MIC	MBC	MIC	MBC	MIC	MBC	MIC
ATCC 43300	MRSA	500	500	500	500	>500	>500	RIF = 0.04
ATCC 25923	MSSA	500	500	500	500	500	>500	RIF = 0.08
ATCC BAA-977	MSSA	500	500	500	500	>500	>500	RIF = 0.08
SAU-UIMY-44	MDR, MRSA, VRSA	500	>500	500	>500	>500	>500	TET = 0.5
SAU-UIMY-31	MSSA	500	500	250	500	250	250	RIF = 0.08
SAU-UIMY-24	MDR, MRSA, VSSA	250	500	250	500	500	500	TET = 0.5
SAU-UIMY-5	MDR, MRSA	250	250	250	250	250	250	RIF = 0.04
SAU-UIMY- 1	XDR, MRSA, VISA	**125**	250	**125**	250	>500	>500	AMK = 16
SAU-UIMY-26	MDR, MSSA, VISA	**125**	**125**	**125**	**125**	>500	>500	TET = 0.5
SAU-UIMY-10	MDR, MRSA, VSSA	**125**	**125**	**125**	**125**	250	500	TET = 0.5
SAU-UIMY-39	MDR, MSSA, VSSA	**125**	**125**	**125**	250	250	250	TET = 0.5
SAU-UIMY-16	MDR, MRSA, VSSA	**125**	**125**	**62.5**	**125**	**125**	250	TET = 0.5
		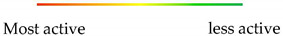						

Car: carvacrol; Thy: thymol; Ses: sesquiterpene; MIC: minimal inhibitory concentration and MBC: minimal bactericidal concentration; MSSA: Methicillin-Susceptible *Staphylococcus aureus*; MRSA: Methicillin-Resistant *Staphylococcus aureus*; MDR: MultiDrug-Resistant; VSSA: Vancomycin-Susceptible *Staphylococcus aureus*; VISA: Vancomycin-Intermediate *Staphylococcus aureus*; VRSA: Vancomycin-Resistant *Staphylococcus aureus*; XDR: Extensively Drug-Resistant; RIF: Rifampin; TET: Tetracycline; AMK: Amikacin. MIC and MBC values are expressed as μg/mL.

**Table 3:** Activity of the three chemotypes of the *L. origanoides* EO on *S. aureus* biofilm formation.

*S. aureus* Strain	Origin	IC_50_ (µg/mL)				Biofilm Biomass (OD Values)
		*Lippia origanoides* Chemotypes			Positive Control	
		Car	Thy	Sesq	EDTA	
ATCC 43300	-	90.0 ± 1.9 ^a,b^	149.3 ± 1.9 ^a,b^	156.9 ± 2.1 ^a,b^	79.5 ± 2.9	0.728 ± 0.08
SAU-UIMY-26	Surgical wound	379.0 ± 7.0 ^e^	568.3 ± 20.9 ^f^	706.6 ± 173.5 ^g^	406.9 ± 1.2	0.198 ± 0.01
SAU-UIMY-44	Blood	228.3 ± 7.5 ^b,c,d^	291.7 ± 14.9 ^b,c,d,e^	684.86 ± 90.4 ^f,g^	458.1 ± 3.3	0.324 ± 0.03
SAU-UIMY-16	Bronchial secretion	213.8 ± 5.4 ^b,c,d^	192.8 ± 7.7 ^b,c,d^	659.3 ± 62.8 ^f,g^	123.9 ± 5.0	0.581 ± 0.06
SAU-UIMY-24	Surgical wound	196.9 ± 6.8 ^b,c,d^	198.6 ± 3.3 ^b,c,d^	307.8 ± 12.8 ^d,e^	55.8 ± 2.6	0.407 ± 0.07
SAU-UIMY-10	Urine	169.6 ± 4.5 ^b,c^	162.4 ± 7.4 ^b,c^	350.0 ± 2.0 ^d,e^	257.8 ± 4.6	0.420 ± 0.07
SAU-UIMY-31	Breast abscess	99.3 ± 3.9 ^a,b^	82.4 ± 5.1 ^a,b^	79.1 ± 6.1 ^a,b^	199 ± 1.8	0.902 ± 0.2
SAU-UIMY-1	Bronchial secretion	77.3 ± 4.8 ^a,b^	60.6 ± 1.7 ^a,b^	30.8 ± 1.5 ^a^	84.8 ± 1.8	1.29 ± 0.27
SAU-UIMY-39	Blood	35.5 ± 1.8 ^a,b^	63.3 ± 4.1 ^a,b^	103.2 ± 5.2 ^a,b^	104.8 ± 3.1	0.482 ± 0.08
		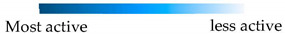				

Car: carvacrol; Thy: thymol; Ses: sesquiterpene; EDTA: ethylendiamine tetraacetic acid; average IC_50_ values (± SD) with the same letter showed non-significant differences in the post hoc Tukey test (*p* < 0.05).

**Table 4:** Spearman rank correlation coefficient values between the IC_50_ of antibiofilm activity and the concentration of the major components in the EO of the three chemotypes of *L. origanoides*.

*S. aureus* Strain	*p*-cymene	eucalyptol	thymol	carvacrol	β-caryophyllene	α-humulene	α+β-selinene	γ+δ-cadinene
SAU-UIMY-1	0.95	−0.95	0.47	0.95	−0.95	−0.95	−0.95	−0.95
SAU-UIMY-31	0.74	−0.74	0.21	0.74	−0.74	−0.74	−0.74	−0.74
SAU-UIMY-16	*−0.53*	*0.53*	−0.90	*−0.53*	*0.53*	*0.53*	*0.53*	*0.53*
SAU-UIMY-10	−0.63	0.63	−0.79	−0.63	0.63	0.63	0.63	0.63
SAU-UIMY-24	−0.74	0.74	−0.69	−0.74	0.74	0.74	0.74	0.74
SAU-UIMY-26	−0.84	0.84	−0.26	−0.84	0.84	0.84	0.84	0.84
SAU-UIMY-39	−0.95	0.95	−0.47	−0.95	0.95	0.95	0.95	0.95
SAU-UIMY-44	−0.95	0.95	−0.47	−0.95	0.95	0.95	0.95	0.95
ATCC 43300	−0.84	0.84	−0.26	−0.84	0.84	0.84	0.84	0.84

Non-significant correlation values > 0.05 are shown in italics for each of the nine analyzed *S. aureus* strains (N = 9). Highlighted in green are significant negative correlations, indicating that a lower IC_50_ is associated with a higher concentration of the component percentage in the EO.

**Table 5:** Origin and drug-resistant profile of *S. aureus* strains.

*S. aureus* Strain	Drug-Resistant Profile	Origin of Clinical Isolate	Susceptible To	Resistant To
**ATCC 43300**	MDR, MRSA	—	DAP, LVX, LZD, MXF, RIF, SNC, SXT, TET, VAN	AMC, AMP, CLI, CRO, ERY, GEN, OXA, PEN, MET, SAM
**ATCC 25923**	MSSA	—	AMC, AMP, CLI, CRO, DAP, ERY, GEN, LVX, LZD, MET, MXF, PEN, OXA, RIF, SAM, SNC, SXT, TET, VAN	—
**ATCC BAA-977**	MSSA	—	CHL, CIP, CLI, CPT, DAP, GEN, LZD, OXA, RIF, SXT, TGC, VAN	ERY, PEN
**SAU-UIMY-39**	MDR, MSSA, VSSA	blood	AMC, CRO, DAP, GEN, LVX, LZD, MET, MXF, OXA, RIF, SAM, SNC, SXT, TET, VAN	AMP, CLI, ERY, PEN
**SAU-UIMY-44**	MDR, MRSA, VRSA	blood	SXT, TET	AMC, AMP, CIP, CLI, CRO, ERY, LVX, MET, MFX, OXA, PEN, RIF, SAM, SNC, VAN
**SAU-UIMY-31**	MSSA	breast abscess	AMC, CLI, CRO, DAP, ERY, GEN, LVX, LZD, MET, MXF, OXA, RIF, SAM, SNC, SXT, TET, VAN	AMP, PEN
**SAU-UIMY-16**	MDR, MRSA, VSSA	bronchial secretion	DAP, GEN, LZD, SNC, SXT, TET, VAN	AMC, AMP, CLI, CRO, ERY, LVX, MET, MXF, OXA, PEN, RIF, SAM, SNC
**SAU-UIMY-1**	XDR, MRSA, VISA	bronchial secretion	DAP, LZD	AMC, AMP, CIP, CLI, CRO, ERY, LVX, MET, MXF, OXA, PEN, RIF, SAM, SXT, TET
**SAU-UIMY-5**	MDR, MRSA	surgical wound	DAP, GEN, LZD, RIF, SNC, SXT, TET, VAN	AMC, AMP, CIP, CLI, CRO, ERY, LVX, MET, MXF, OXA, PEN, SAM,
**SAU-UIMY-24**	MDR, MRSA, VSSA	surgical wound	CIP, DAP, ERY, GEN, LVX, LZD, MXF, RIF, SNC, SXT, TET, VAN	AMC, AMP, CLI, CRO, MET, OXA, PEN, SAM,
**SAU-UIMY-26**	MDR, MSSA, VISA	surgical wound	AMC, CLI, CRO, DAP, ERY, LVX, LZD, MET, MXF, OXA, RIF, SAM, SXT, TET	AMP, GEN, PEN, SNC,
**SAU-UIMY-10**	MDR, MRSA, VSSA	urine	DAP, SXT, TET, VAN	AMC, AMP, CIP, CLI, CRO, ERY, GEN, LVX, LZD, MET, MXF, OXA, PEN, RIF, SAM, SNC

Abbreviations: SAU-UIMY: clinical isolates from UIMY biobank, AMC: Amoxicillin/Acid clavulanic, AMP: Ampicillin, CHL: Chloramphenicol, CIP: Ciprofloxacin, CLI: Clindamycin, CPT: ceftaroline, CRO: Ceftriaxone, DAP: Daptomycin, ERY: Erythromycin, GEN: Gentamicin, Levofloxacin, LZD: Linezolid, MET: Methicillin, MXF: Moxifloxacin, OXA: Oxacillin, PEN: penicillin, RIF: Rifampicin, SAM: Ampicillin/Sulbactam, SNC: Synercid, SXT: Trimethoprim/Sulfamethoxazole, TET: Tetracycline, TGC: Tigeciclina, VAN: Vancomycin, MDR: multidrug-resistant, MRSA: Methicillin-resistant *Staphylococcus aureus*, MSSA: Methicillin-susceptible *Staphylococcus aureus*, VRSA: Vancomycin-resistant *Staphylococcus aureus*, VSSA: Vancomycin-susceptible *Staphylococcus aureus,* VISA: Vancomycin-intermediate *Staphylococcus aureus.*

## Data Availability

Data are contained within the article.

## References

[1] Wohlleben W., Mast Y., Stegmann E., Ziemert N. (2016). Antibiotic drug discovery. Microb. Biotechnol..

[2] Lee Ventola C. (2015). The antibiotic resistance crisis part 1: Causes and threats. Pharm. Ther..

[3] Prestinaci F., Pezzotti P., Pantosti A. (2015). Antimicrobial resistance: A global multifaceted phenomenon. Pathog. Glob. Health.

[4] Chinemerem Nwobodo D., Ugwu M.C., Oliseloke Anie C., Al-Ouqaili M.T.S., Chinedu Ikem J., Victor Chigozie U., Saki M. (2022). Antibiotic resistance: The challenges and some emerging strategies for tackling a global menace. J. Clin. Lab. Anal..

[5] Mancuso G., Midiri A., Gerace E., Biondo C. (2021). Bacterial antibiotic resistance: The most critical pathogens. Pathogens.

[6] Turner N.A., Sharma-Kuinkel B.K., Maskarinec S.A., Eichenberger E.M., Shah P.P., Carugati M., Holland T.L., Fowler V.G. (2019). Methicillin-Resistant *Staphylococcus aureus*: An overview of basic and clinical research. Nat. Rev. Microbiol..

[7] Yu Z., Tang J., Khare T., Kumar V. (2020). The alarming antimicrobial resistance in ESKAPEE pathogens: Can essential oils come to the rescue?. Fitoterapia.

[8] Kumar V., Yasmeen N., Pandey A., Ahmad Chaudhary A., Alawam A.S., Ahmad Rudayni H., Islam A., Lakhawat S.S., Sharma P.K., Shahid M. (2023). Antibiotic adjuvants: Synergistic tool to combat multi-drug resistant pathogens. Front. Cell. Infect. Microbiol..

[9] Reichling J. (2020). Anti-biofilm and virulence factor-reducing activities of essential oils and oil components as a possible option for bacterial infection control. Planta Med..

[10] Álvarez-Martínez F.J., Barrajón-Catalán E., Herranz-López M., Micol V. (2021). Antibacterial plant compounds, extracts and essential oils: An updated review on their effects and putative mechanisms of action. Phytomedicine.

[11] Brożyna M., Paleczny J., Kozłowska W., Chodaczek G., Dudek-Wicher R., Felińczak A., Gołębiewska J., Górniak A., Junka A. (2021). The antimicrobial and antibiofilm in vitro activity of liquid and vapour phases of selected essential oils against *Staphylococcus aureus*. Pathogens.

[12] Romanescu M., Oprean C., Lombrea A., Badescu B., Teodor A., Constantin G.D., Andor M., Folescu R., Muntean D., Danciu C. (2023). Current state of knowledge regarding who high priority pathogens—Resistance mechanisms and proposed solutions through candidates such as essential oils: A systematic review. Int. J. Mol. Sci..

[13] Kintzios S.E., Kintzios S.E. (2002). Profile of the multifaceted prince of the herbs. Oregano the Genera Origanum and Lippia.

[14] O’Leary N., Denham S.S., Salimena F., Múlgura M.E. (2012). Species delimitation in *Lippia* section *Goniostachyum* (verbenaceae) using the phylogenetic species concept. Bot. J. Linn. Soc..

[15] Atlas de Las Plantas de La Medicina Tradicional Mexicana. http://www.medicinatradicionalmexicana.unam.mx/apmtm/index.html.

[16] Calvo-Irabien L.M. (2018). Native mexican aromatic flora and essential oils: Current research status, gaps in knowledge and agro-industrial potential. Ind. Crops. Prod..

[17] Siqueira-Lima P.S., Passos F.R.S., Lucchese A.M., Menezes I.R.A., Coutinho H.D.M., Lima A.A.N., Zengin G., Quintans J.S.S., Quintans-Júnior L.J. (2019). Central nervous system and analgesic profiles of *Lippia* genus. Rev. Bras. Farm..

[18] Candelaria-Dueñas S., Serrano-Parrales R., Ávila-Romero M., Meraz-Martínez S., Orozco-Martínez J., Ávila-Acevedo J.G., García-Bores A.M., Cespedes-Acuña C.L., Peñalosa-Castro I., Hernandez-Delgado T. (2021). Evaluation of the antimicrobial activity of some components of the essential oils of plants used in the traditional medicine of the Tehuacán-Cuicatlán valley, Puebla, México. Antibiotics.

[19] Zapién-Chavarría K.A., Plascencia-Terrazas A., Venegas-Ortega M.G., Varillas-Torres M., Rivera-Chavira B.E., Adame-Gallegos J.R., González-Rangel M.O., Nevárez-Moorillón G.V. (2019). Susceptibility of multidrug-resistant and biofilm-forming uropathogens to mexican oregano essential oil. Antibiotics.

[20] Gómez-Sequeda N., Cáceres M., Stashenko E.E., Hidalgo W., Ortiz C. (2020). Antimicrobial and antibiofilm activities of essential oils against *Escherichia coli* o157: H7 and methicillin-resistant *Staphylococcus aureus* (MRSA). Antibiotics.

[21] Dhifi W., Bellili S., Jazi S., Bahloul N., Mnif W. (2016). Essential oils’ chemical characterization and investigation of some biological activities: A critical review. Medicines.

[22] Feyaerts A.F., Mathé L., Luyten W., De Graeve S., Van Dyck K., Broekx L., Van Dijck P. (2018). Essential oils and their components are a class of antifungals with potent vapour-phase-mediated anti-candida activity. Sci. Rep..

[23] El-Tarabily K.A., El-Saadony M.T., Alagawany M., Arif M., Batiha G.E., Khafaga A.F., Elwan H.A.M., Elnesr S.S., El-Hack M.E.A. (2021). Using essential oils to overcome bacterial biofilm formation and their antimicrobial resistance. Saudi J. Biol. Sci..

[24] Bassolé I.H.N., Juliani H.R. (2012). Essential oils in combination and their antimicrobial properties. Molecules.

[25] Chouhan S., Sharma K., Guleria S. (2017). Antimicrobial activity of some essential oils—Present status and future perspectives. Medicines.

[26] Chraibi M., Farah A., Elamin O., Iraqui H., Fikri-Benbrahim K. (2020). Characterization, antioxidant, antimycobacterial, antimicrobial effects of *Moroccan rosemary* essential oil, and its synergistic antimicrobial potential with carvacrol. J. Adv. Pharm. Technol. Res..

[27] Trindade H., Pedro L.G., Figueiredo A.C., Barroso J.G. (2018). Chemotypes and terpene synthase genes in *Thymus* genus: State of the art. Ind. Crops Prod..

[28] Stashenko E., Martínez J., Ruiz C., Arias G., Duran C., Salgar W., Cala M. (2010). *Lippia origanoides* chemotype differentiation based on essential oil GC-MS and principal components analysis. J. Sep. Sci..

[29] Calvo-Irabién L.M., Parra-Tabla V., Acosta-Arriola V., Escalante-Erosa F., Díaz-Vera L., Dzib G.R., Peña-Rodríguez L.M. (2014). Phytochemical diversity of the essential oils of mexican oregano (*Lippia graveolens* Kunth) populations along an edapho-climatic gradient. Chem. Biodivers..

[30] Ribeiro A.F., Andrade E.H.A., Salimena F.R.G., Maia J.G.S. (2014). Circadian and seasonal study of the cinnamate chemotype from *Lippia origanoides* Kunth. Biochem. Syst. Ecol..

[31] Méndez González M.E., Durán García R., Borges Argáez R., Peraza Sánchez S., Dorantes Euan A., Tapia Muñoz J.L. (2012). Flora Medicinal: De los Mayas Peninsulares.

[32] Fimbres-García J.O., Flores-Sauceda M., Othon-Díaz E.D., García-Galaz A., Tapia-Rodríguez M.R., Silva-Espinoza B.A., Ayala-Zavala J.F. (2022). Facing resistant bacteria with plant essential oils: Reviewing the oregano case. Antibiotics.

[33] Hao Y., Kang J., Yang R., Li H., Cui H., Bai H., Tsitsilin A., Li J., Shi L. (2022). Multidimensional exploration of essential oils generated via eight oregano cultivars: Compositions, chemodiversities, and antibacterial capacities. Food Chem..

[34] Martínez A., Manrique-Moreno M., Klaiss-Luna M.C., Stashenko E., Zafra G., Ortiz C. (2021). Effect of essential oils on growth inhibition, biofilm formation and membrane integrity of *Escherichia coli* and *Staphylococcus aureus*. Antibiotics.

[35] Martínez A., Stashenko E.E., Sáez R.T., Zafra G., Ortiz C. (2023). Effect of essential oil from *Lippia origanoides* on the transcriptional expression of genes related to quorum sensing, biofilm formation, and virulence of *Escherichia coli* and *Staphylococcus aureus*. Antibiotics.

[36] Ben Abdallah F., Lagha R., Gaber A. (2020). Biofilm inhibition and eradication properties of medicinal plant essential oils against methicillin-resistant *Staphylococcus aureus* clinical isolates. Pharmaceuticals.

[37] Kwiecinski J.M., Jacobsson G., Horswill A.R., Josefsson E., Jin T. (2019). Biofilm formation by *Staphylococcus aureus* clinical isolates correlates with the infection type. Infect. Dis..

[38] Tan L., Huang Y., Shang W., Yang Y., Peng H., Hu Z., Wang Y., Rao Y., Hu Q., Rao X. (2022). Accessory gene regulator (*agr*) allelic variants in cognate *Staphylococcus aureus* strain display similar phenotypes. Front. Microbiol..

[39] dos Santos Rodrigues J.B., de Carvalho R.J., de Souza N.T., de Sousa Oliveira K., Franco O.L., Schaffner D., de Souza E.L., Magnani M. (2017). Effects of oregano essential oil and carvacrol on biofilms of *Staphylococcus aureus* from food-contact surfaces. Food Control..

[40] Wu X., Wang H., Xiong J., Yang G.-X., Hu J.-F., Zhu Q., Chen Z. (2024). *Staphylococcus aureus* biofilm: Formulation, regulatory, and emerging natural products-derived therapeutics. Biofilm.

[41] Kim Y., Shin M., Kang J., Kang D. (2022). Effect of sub-lethal treatment of carvacrol and thymol on virulence potential and resistance to several bactericidal treatments of *Staphylococcus aureus*. J. Food Saf..

[42] Knowles J.R., Roller S., Murray D.B., Naidu A.S. (2005). Antimicrobial action of carvacrol at different stages of dual-species biofilm development by *Staphylococcus aureus* and *Salmonella enterica* serovar *Typhimurium*. Appl. Environ. Microbiol..

[43] Nostro A., Roccaro A.S., Bisignano G., Marino A., Cannatelli M.A., Pizzimenti F.C., Cioni P.L., Procopio F., Blanco A.R. (2007). Effects of oregano, carvacrol and thymol on *Staphylococcus aureus* and *Staphylococcus epidermidis* biofilms. J. Med. Microbiol..

[44] Miladi H., Zmantar T., Chaabouni Y., Fedhila K., Bakhrouf A., Mahdouani K., Chaieb K. (2016). Antibacterial and efflux pump inhibitors of thymol and carvacrol against food-borne pathogens. Microb. Pathog..

[45] Ahmad A., Van Vuuren S., Viljoen A. (2014). Unravelling the complex antimicrobial interactions of essential oils—The case of *Thymus vulgaris* (Thyme). Molecules.

[46] Cáceres M., Hidalgo W., Stashenko E., Torres R., Ortiz C. (2020). Essential oils of aromatic plants with antibacterial, anti-biofilm and anti-quorum sensing activities against pathogenic bacteria. Antibiotics.

[47] Oliveira Ribeiro S., Fontaine V., Mathieu V., Abdesselam Z., Dominique B., Caroline S., Florence S. (2021). Antibacterial activities of homemade matrices miming essential oils compared to commercial ones. Antibiotics.

[48] Dahham S.S., Tabana Y.M., Iqbal M.A., Ahamed M.B.K., Ezzat M.O., Majid A.S.A., Majid A.M.S.A. (2015). The anticancer, antioxidant and antimicrobial properties of the sesquiterpene β-caryophyllene from the essential oil of *Aquilaria crassna*. Molecules.

[49] Thi Huong L., Chung N., Duc D., Dai D., Pham The H., Ninh The S. (2023). Essential oil of *Syzygium boisianum* (Gagnep.) Merr. & L.M.Perry: Chemical compositions, antimicrobial activity, and molecular docking. Vietnam J. Chem..

[50] Huong L.T., Sam L.N., Dai D.N., Pham T.V., Son N.T. (2022). Essential oils of two ginger plants *Newmania orthostachys* N.S. Lý & Škorničk. and N. Serpens N.S. Lý & Škorničk.: Chemical compositions and antimicrobial activity. J. Essent. Oil Bear. Plants.

[51] Martínez-Natarén D.A., Parra-Tabla V., Ferrer-Ortega M.M., Calvo-Irabién L.M. (2014). Genetic diversity and genetic structure in wild populations of mexican oregano (*Lippia graveolens* H.B.K.) and its relationship with the chemical composition of the essential oil. Plant Syst. Evol..

[52] Adams R.P. (2007). Identification of Essential Oil Components by Gas Chromatography/Mass Spectrometry.

[53] Babushok V.I., Linstrom P.J., Zenkevich I.G. (2011). Retention indices for frequently reported compounds of plant essential oils. J. Phys. Chem. Ref. Data.

[54] Zellner B. (2008). d’Acampora; Bicchi, C.; Dugo, P.; Rubiolo, P.; Dugo, G.; Mondello, L. Linear retention indices in gas chromatographic analysis: A review. Flavour Fragr. J..

[55] Uc-Cachón A.H., de Dzul-Beh A.J., Palma-Pech G.A., Jiménez-Delgadillo B., Flores-Guido J.S., Gracida-Osorno C., Molina-Salinas G.M. (2021). Antibacterial and antibiofilm activities of mayan medicinal plants against methicillin-susceptible and -resistant strains of *Staphylococcus aureus*. J. Ethnopharmacol..

[56] de Jesús Dzul-Beh A., Uc-Cachón A.H., González-Sánchez A.A., Dzib-Baak H.E., Ortiz-Andrade R., Barrios-García H.B., Jiménez-Delgadillo B., Molina-Salinas G.M. (2023). Antimicrobial potential of the mayan medicine plant *Matayba oppositifolia* (A. Rich.) britton against antibiotic-resistant priority pathogens. J. Ethnopharmacol..

[57] Molina-Salinas G.M., Ramos-Guerra M.C., Vargas-Villarreal J., Mata-Cárdenas B.D., Becerril-Montes P., Said-Fernández S. (2006). Bactericidal activity of organic extracts from *Flourensia cernua* DC against strains of *Mycobacterium tuberculosis*. Arch. Med. Res..

[58] Loges L.A., Silva D.B., Paulino G.V.B., Landell M.F., Macedo A.J. (2020). Polyketides from marine-derived *Aspergillus welwitschiae* inhibit *Staphylococcus aureus* virulence factors and potentiate vancomycin antibacterial activity in vivo. Microb. Pathog..

